# Internal Teat Sealant as an Alternative to Intramammary Antibiotics at Dry-Off in Low-Risk Dairy Cows: Effects on Udder Health, Milk Yield, Antimicrobial Use, and Economic Outcomes

**DOI:** 10.3390/ani16121772

**Published:** 2026-06-08

**Authors:** Ionela Delia Ut, Daniel Ionut Berean, Liviu Marian Bogdan, Simona Ciupe, Sidonia Gog-Bogdan

**Affiliations:** 1 Department of Reproduction, Faculty of Veterinary Medicine, University of Agricultural Sciences and Veterinary Medicine, Calea Manastur 3–5, 400372 Cluj-Napoca, Romania; ionela-delia.ut@student.usamvcluj.ro (I.D.U.); liviu.bogdan@usamvcluj.ro (L.M.B.); simona.ciupe@usamvcluj.ro (S.C.); 2 Department of Surgery, Anesthesiology and Intensive Care Faculty of Veterinary Medicine, University of Agricultural Sciences and Veterinary Medicine, Calea Manastur 3–5, 400372 Cluj-Napoca, Romania; sidonia.bogdan@usamvcluj.ro

**Keywords:** selective dry cow therapy, intramammary infection, somatic cell count, mastitis control, antimicrobial stewardship, Romanian dairy farms

## Abstract

Selective dry cow therapy (SDCT) has emerged as a key strategy to reduce antimicrobial use in dairy production while maintaining udder health. This study aimed to evaluate the feasibility and impact of implementing SDCT in Romanian dairy farms by comparing low-risk cows treated with internal teat sealant only (ITS) at dry-off with low-risk cows treated with intramammary antibiotics at dry-off. A prospective field study was conducted on two commercial dairy herds, including 87 cows classified based on somatic cell count (SCC) and differential SCC (DSCC), and compared with a historical cohort of 37 cows. Udder health parameters, milk yield during the first 100 days in milk (DIM), antimicrobial use, and economic outcomes were evaluated. No significant differences were observed between groups in terms of postpartum intramammary infections, somatic cell score, DSCC, or clinical mastitis incidence. Milk yield during early lactation was also not affected by treatment. The ITS-only strategy resulted in a substantial reduction in antimicrobial use (−88.8% per cow) without significant differences in total economic costs. Farm-related differences highlighted the influence of management conditions on outcomes. These findings indicate that, in low-risk cows, SDCT using ITS alone is a safe and effective alternative to antibiotic treatment and support the feasibility of implementing SDCT under Romanian dairy production conditions as a sustainable strategy to promote the targeted and prudent use of antimicrobials while reducing unnecessary antibiotic exposure in dairy herds. However, given the limited number of herds and animals included, further studies are needed to confirm these promising findings under a broader range of production conditions.

## Simple Summary

Reducing the use of antibiotics in dairy farming is an important goal for both animal health and public health. Traditionally, many dairy farms have used antibiotics at dry-off to eliminate existing intramammary infections acquired during lactation and to provide protection against new infections during the early dry period, including for cows without clinical signs of disease. This study investigated whether low-risk cows can be safely managed without antibiotics by using a physical barrier called an internal teat sealant, which protects the udder during the dry period. The study was conducted on two commercial dairy farms in Romania and included cows identified as healthy based on milk quality indicators. The results showed that cows treated only with the sealant had similar udder health, milk production, and risk of mastitis compared with cows treated with antibiotics. At the same time, the use of antibiotics was greatly reduced without increasing overall costs. Differences between farms highlighted the importance of management conditions in influencing outcomes. These findings suggest that, in well-managed herds with low disease risk, antibiotics can be reduced without negative effects, supporting more sustainable dairy farming practices and helping to limit antimicrobial resistance.

## 1. Introduction

Mastitis is a multifactorial disease resulting from the complex interaction between microbial pathogens, host-related factors, environmental conditions, and management practices [1], representing a major health issue in dairy herds and adversely affecting both animal health and welfare [2]. From an economic perspective, mastitis leads to substantial losses, driven by direct costs associated with diagnosis, treatment, and the disposal of milk unfit for human consumption, as well as indirect costs related to reduced milk production and premature culling of affected animals [3].

The dry period represents a critical stage for maintaining udder health and ensuring optimal performance in the subsequent lactation [4]. During this interval, the mammary gland undergoes a physiological involution process, characterized by increased secretion of antimicrobial compounds such as lactoferrin and lysozyme, as well as the formation of a keratin plug within the teat canal [5]. However, the risk of new intramammary infections (IMIs) remains elevated, particularly during the early dry period, when the keratin barrier is not yet fully established and the natural flushing effect associated with regular milking is absent [6,7]. The rate of new infections decreases as involution progresses, but rises again in the period close to calving [8]. IMIs that develop or persist during the dry period are a major determinant of clinical mastitis (CM) in the subsequent lactation [9,10,11,12], with some studies indicating that more than 50% of early-lactation CM cases originate during this stage [13,14,15].

Therefore, in order to treat existing IMIs at dry-off and to prevent the occurrence of new IMIs during the dry period [16], intramammary antibiotic therapy at dry-off, commonly referred to as blanket dry cow therapy (BDCT), was introduced in the late 1960s as part of the National Mastitis Control Plan [17,18]. This strategy involves the intramammary administration of long-acting antimicrobials to all cows at dry-off, regardless of their infection status. Its widespread implementation in the dairy industry over several decades [17,18] has significantly contributed to improvements in udder health worldwide, particularly through the reduction of contagious mastitis and a shift in the etiological profile toward a predominance of environmental pathogens [19].

However, the widespread implementation of BDCT has been associated with a substantial use of antimicrobials. In 2009, intramammary treatments accounted for more than 60% of total antimicrobial use in Dutch dairy farms, with approximately two-thirds attributed to BDCT [20]. Similarly, in the United States, the application of BDCT has been estimated to result in the annual use of approximately 11 tons of antibiotics [21]. In this context, concerns regarding the contribution of routine antibiotic use at dry-off to the emergence and spread of antimicrobial resistance have led to a reevaluation of the sustainability of this practice [22].

Against this background, European policies have established clear targets for reducing antimicrobial use in the livestock sector. According to Clabby et al. [23], the European Union (EU) has committed to a 50% reduction in antimicrobial consumption in animal production systems by 2030, in response to the World Health Organization’s global call for optimizing antibiotic use. In line with these objectives, Regulation (EU) 2019/6 on veterinary medicinal products, applicable since January 2022, states that antimicrobials should not be used routinely and that prophylactic or metaphylactic administration, including intramammary use, must be strictly limited [24]. Within the One Health framework, achieving a balance between effective control of IMIs, essential for animal health and welfare, and the need to reduce antimicrobial use has therefore become a key priority [25].

Consequently, in herds characterized by a low prevalence of contagious mastitis and consistently low herd SCC levels based on routine milk recording (<200,000 cells/mL), antibiotic treatment at dry-off may not be necessary for all animals, although selection criteria may vary between herds and management systems. Under these conditions, a targeted approach to antimicrobial use, limited to infected cows and those at increased risk of developing IMIs at the end of lactation, has emerged as an alternative, commonly referred to as selective dry cow therapy (SDCT) [26]. This strategy can be applied using different approaches, either at the cow level or at the quarter level [27].

It is widely acknowledged that the successful implementation of this therapeutic approach depends on appropriate cow selection together with adequate management during the dry period and around calving [28], as failure to treat cows affected by major pathogens may have negative consequences for udder health and farm economic performance. Therefore, practical, accessible, and cost-effective methods are required to reliably distinguish cows or quarters that would likely benefit from antimicrobial treatment from those that can be left untreated [29].

Bacteriological culture remains the reference (“gold standard”) method; however, in practice, alternative approaches that are faster or applicable on-farm are also used, including rapid culture systems [27,30], strip tests for esterase activity as an indirect indicator of SCC, milk conductivity measurements, and assays for enzymes such as lactate dehydrogenase and N-acetyl-β-D-glucosaminidase [31,32], as well as the California Mastitis Test [33]. In addition, selection of cows for dry-off therapy can be based on decision algorithms integrating mastitis history, including the number of CM cases during the current lactation and monthly SCC records [30,34,35]. More recently, differential somatic cell count (DSCC) has been introduced as an additional indicator based on the proportion of major immune cell populations in milk [36]. DSCC represents the combined percentage of polymorphonuclear neutrophils (PMN) and lymphocytes within the total SCC and tends to increase with rising SCC values, with elevated DSCC levels often associated with the presence of IMI [36]. Compared with total SCC, the proportion of neutrophils and lymphocytes may more accurately reflect the inflammatory status of the mammary gland, as these cell types are typically increased in milk from cows with high SCC or mastitis [37,38]. Furthermore, the combined use of DSCC and SCC has been shown to improve the accuracy of identifying infected animals for SDCT implementation while also representing a practical and cost-effective approach in herds with regular SCC monitoring, typically performed at intervals of approximately 4–8 weeks. In such systems, where SCC records are routinely available, DSCC may represent a useful complementary indicator for SDCT decision-making [3,25,39,40].

With regard to the impact of SDCT on udder health, recent evidence indicates that this strategy performs comparably to BDCT in terms of preventing new IMIs during the dry period, as well as in reducing the incidence of postpartum CM and maintaining SCC levels during early lactation [4,5,26,30,31,35,41,42,43,44]. Furthermore, no significant differences have been reported in milk yield (MY) or culling rates within the first 120 days in milk (DIM), regardless of whether SDCT was performed at the quarter level based on rapid culture or at the cow level using predictive algorithms, provided that internal teat sealants (ITS) were used [26,27,30,35,41,42].

Although SDCT has been widely adopted in certain countries, its implementation remains limited at a global level, with blanket dry cow therapy still predominating in many production systems [45]. Despite encouraging results reported in Western European production systems, uncertainties remain regarding the feasibility and performance of SDCT under different regional management conditions and breed structures. Most available studies originate from Western Europe and have been conducted in intensive farming systems, resulting in limited evidence on SDCT application in Eastern European dairy farms. In particular, information remains scarce regarding SDCT implementation under Romanian dairy production systems, where herd structure, management practices, adoption of milk recording programs, and dry-off practices may differ from those commonly reported in Western Europe, in addition to the predominance of Romanian Spotted cattle.

In this context, the present study did not aim to directly compare BDCT and SDCT at the herd level, but rather to evaluate a key component of SDCT, namely the management of cows classified as low-risk for IMIs at dry-off. The objective of the study was to assess whether the use of ITS alone in cows considered healthy is comparable to intramammary antibiotic treatment in terms of postpartum udder health and milk production, as well as to quantify the impact of this strategy on antimicrobial use and economic performance. By generating field data under Romanian production conditions, the study also sought to contribute locally relevant evidence to support informed decision-making regarding SDCT implementation in dairy practice.

The study hypothesis was that, in cows considered uninfected at dry-off, the exclusive use of ITS would be non-inferior to intramammary antibiotic treatment with respect to postpartum udder health parameters, while significantly reducing antimicrobial use.

## 2. Materials and Methods

### 2.1. Farms and Management Conditions

A prospective field study was conducted between May 2025 and April 2026 on two commercial dairy farms in Romania. Both herds consisted exclusively of Romanian Spotted dairy cows and were enrolled in the Dairy Herd Improvement (DHI) milk recording and milk quality monitoring program coordinated by Someș Arieș Agricultural Cooperative (Cluj County, Romania), in which productive performance and milk quality parameters were recorded at regular intervals of approximately four to six weeks. Before study initiation, neither herd had experience with SDCT or the use of ITS, and dry-off management relied exclusively on conventional antibiotic-based protocols. Romanian dairy farming is characterized by considerable heterogeneity in herd size, productivity, and adoption of modern herd-management practices compared with intensive Western European dairy systems. Average milk yield remains below that reported in many Western European countries, and dairy production is frequently fragmented into smaller holdings, highlighting the importance of evaluating SDCT under local production conditions [46,47].

Farm 1 (F1) included 63 lactating cows with an average daily milk yield of 26.4 L per cow, whereas Farm 2 (F2) comprised 51 lactating cows with an average daily milk yield of 20.1 L per cow. The two farms operated under comparable management conditions. Cows were housed in free-stall systems with access to individual resting areas and common feeding spaces. Lactating cows remained continuously housed indoors, whereas during the summer period, heifers and dry cows had access to pasture in both farms. Feeding regimens were similar between farms and consisted of corn silage, alfalfa hay, straw, and protein concentrates supplemented with minerals and vitamins, administered as a total mixed ration (TMR). During the dry period, cows followed routine farm-specific feeding management, consisting primarily of pasture access during summer and coarse forages during the colder season under indoor housing conditions. Although energy balance was not directly assessed, both farms applied comparable nutritional management practices during the dry period. Milking was performed twice daily in conventional milking parlors equipped with hygiene systems. Pre-milking udder hygiene consisted of washing and drying of teats with individual towels in both farms; in F1, this protocol additionally included predipping. Dry-off procedures were carried out according to standard dairy farm practices, consisting of gradual cessation of milking through progressive reduction in milking frequency. The dry period length was 60.8 ± 6.8 days (range 49–76 days) in F1 and 58.9 ± 5.8 days (range 50–71 days) in F2.

### 2.2. Study Design and Animal Selection

The inclusion of the two farms in the study was conditioned by maintaining BMSCC values ≤ 250,000 cells/mL at each routine bulk milk recording over a minimum period of five consecutive months prior to study initiation, consistent with previous SDCT studies using this threshold to identify herds with favorable udder health status and suitability for SDCT implementation [26,41]. During the prospective study period (May 2025–April 2026), all lactating cows reaching dry-off within this interval were evaluated, comprising 51 cows in F1 and 40 cows in F2 (total *n* = 91).

Cows were included if SCC data were available for the last three months prior to dry-off, obtained from DHI records, and if they did not present major systemic diseases at the time of inclusion. Animals that did not meet these criteria or that had received systemic antibiotic treatments in the period immediately preceding dry-off were excluded (2 in F1 and 2 in F2). After applying the inclusion and exclusion criteria, a total of 87 cows (F1: *n* = 49; F2: *n* = 38) were eligible for further analysis.

For comparison, a historical cohort was used, derived from a previous study conducted in the same farms (November 2024–June 2025), which investigated the bacterial flora associated with the dry period and its relationship with SCC and DSCC [48]. From this cohort, cows were retrospectively selected based on the availability of complete SCC data for the three months prior to dry-off, as well as postpartum data up to 100 DIM, including MY, SCC, occurrence of CM, antimicrobial use (AMU), and economic performance, and meeting the same inclusion criteria. This resulted in a total of 37 cows (F1: *n* = 17; F2: *n* = 20), belonging to the same population but evaluated in different lactation cycles.

The cow was considered the unit of analysis, based on considerations of biological relevance, practical feasibility, and economic significance. Accordingly, cows with one blind or non-functional quarter were not excluded from the study. In total, four cows with three functional quarters were included, as SCC, DSCC, milk yield, and treatment allocation were evaluated at the cow level.

An a priori sample size or power calculation was not performed. Instead, all cows meeting the inclusion criteria and reaching dry-off during the study period in the enrolled herds were included consecutively under commercial farm conditions.

### 2.3. Risk Classification and Group Allocation

Cows were classified for dry-off therapy using an algorithm based on SCC and DSCC parameters. Three indicators were considered: the mean SCC over the last three months of lactation prior to dry-off, calculated from DHI records, the SCC value at the last test prior to dry-off, and the DSCC value measured at the same last test prior to dry-off. Thresholds used for classification were set at 200,000 cells/mL for SCC, a widely accepted cutoff for identifying cows at increased risk of IMI [49], and 65% for DSCC. The DSCC threshold was established based on a previous study conducted in the same farms, in which the bacterial flora associated with IMIs and its relationship with DSCC values were characterized [48].

Cows were defined as low-risk if they met all of the following criteria: mean SCC in the last three months prior to dry-off ≤200,000 cells/mL, SCC at the last test prior to dry-off ≤200,000 cells/mL, and DSCC at the last test prior to dry-off ≤65%. Cows were classified as high-risk if they met at least one of the following criteria: mean SCC in the last three months prior to dry-off >200,000 cells/mL, SCC at the last test prior to dry-off >200,000 cells/mL, or DSCC at the last test prior to dry-off >65%. Thus, the classification algorithm followed a conservative approach, whereby the presence of any indicator suggestive of a potential IMI resulted in classification as high-risk. The SCC–DSCC combination was applied as a decision-support approach intended to minimize the risk of leaving potentially infected cows untreated at dry-off rather than as a standalone diagnostic test.

The same classification criteria were retrospectively applied to cows from the previous study used to derive the historical cohort. Only cows meeting the low-risk criteria were selected from this cohort, ensuring that comparisons in the present study were performed between healthy animals with similar baseline characteristics.

In the prospective study, cows classified as high-risk received intramammary antibiotic therapy at dry-off, whereas low-risk cows were treated exclusively with an ITS. The performance of this strategy was evaluated by comparing low-risk cows treated with ITS with low-risk cows from the historical cohort that had received intramammary antibiotics at dry-off.

**Figure 1:**
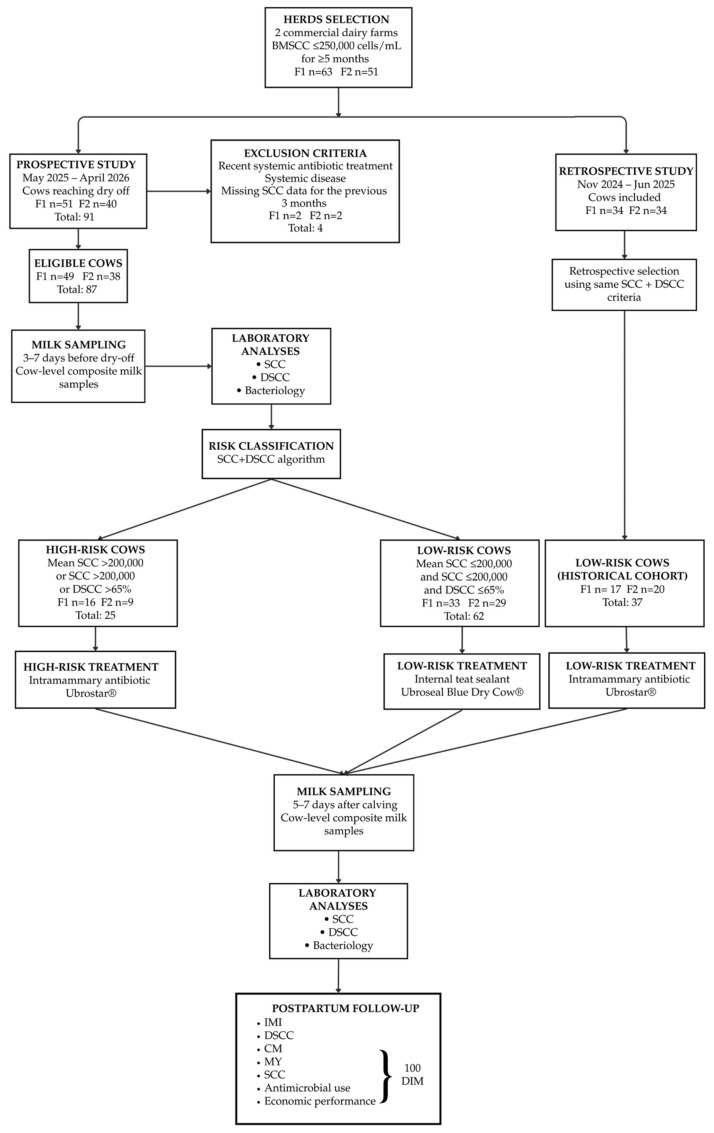
Flow diagram of the study design. Two commercial dairy herds meeting the inclusion criterion of bulk milk somatic cell count (BMSCC ≤ 250,000 cells/mL for at least five consecutive months) were enrolled. Cows dried off during the prospective study (May 2025–April 2026) were classified as high- or low-risk for intramammary infection based on SCC and DSCC thresholds. Low-risk cows received an internal teat sealant, while high-risk cows were treated with intramammary antibiotics. A historical cohort of low-risk cows treated with antibiotics at dry-off (November 2024–June 2025) was included for comparison. Milk samples were collected before dry-off and 5–7 days after calving for SCC, DSCC, and bacteriological analysis to assess IMI. Postpartum follow-up up to 100 DIM included SCC, CM, MY, antimicrobial use, and economic performance. Abbreviations: F1 = Farm 1; F2 = Farm 2; n = number of cows; SCC = somatic cell count; DSCC = differential somatic cell count; IMI = intramammary infection; CM = clinical mastitis; MY = milk yield; DIM = days in milk; BMSCC = bulk milk somatic cell count.

The overall study design, including herd selection, cow inclusion and exclusion criteria, risk classification, and group allocation, is summarized in Figure 1.

### 2.4. Sample Collection and Laboratory Analysis

Milk samples were collected at two time points: 3–7 days prior to dry-off and 5–7 days after calving. The postpartum sampling time was selected to evaluate IMI status and early udder health following the dry period under comparable conditions between groups. Although SCC values may remain physiologically elevated during the early postpartum period, all cows were sampled within the same interval, and SCC assessment was further complemented by DHI-recorded SCC values during the first 100 DIM to provide a broader evaluation of udder health beyond the immediate postpartum phase. Sampling was performed in the morning, before milking, at the cow level (composite milk samples). For each cow, two samples were collected at each time point: one for the determination of SCC and DSCC, and one for microbiological analysis. Samples for SCC and DSCC determination were collected using a milk meter (Melasty, İzmir, Türkiye), a standardized device for measuring milk yield and composition, which enables the collection of a representative milk sample through proportional sampling of milk flow from all four quarters throughout the entire milking process, thereby minimizing variation associated with manual sampling at the beginning or end of milking. These samples were placed in sterile containers without preservatives and transported at a temperature of 4–6 °C to the laboratory within 2 h after collection.

Samples intended for microbiological analysis were collected aseptically, following the protocol recommended by the National Mastitis Council [50]. Teat preparation included removal of dirt and debris by brushing, forestripping (discarding the first streams of milk), followed by disinfection of each teat using an iodine-based solution (KerbaWasch^®^ 2%, Kerbl, Buchbach, Germany), which was allowed to act for 30 s. The teats were then dried using individual paper towels. Immediately prior to sampling, the teat end and barrel were disinfected with sterile gauze soaked in 70% isopropyl alcohol. Milk samples were collected under hygienic conditions using disposable nitrile gloves. Subsequently, 10–20 mL of milk was collected from each quarter into sterile plastic containers for standard aerobic bacterial culture. All samples were appropriately labeled, and sample identification numbers were linked to individual cow identities. Subsequently, samples were stored on ice and transported promptly to the laboratory for processing.

Samples for SCC and DSCC determination, as well as microbiological samples, were processed at the Laboratory of the Milk Quality Control Foundation in Cluj-Napoca, Romania.

#### 2.4.1. Determination of SCC and DSCC

SCC and DSCC were determined using an automated Fossomatic™ system (FOSS, Hillerød, Denmark), based on flow cytometry and in accordance with International Dairy Federation (IDF) standards. DSCC determination was performed according to the method described by Damm et al. [36], which allows the identification, within a milk sample, of macrophages (MAC) and the combined population of PMN and lymphocytes (LYM). DSCC is expressed as the combined proportion (%) of PMN and LYM relative to the total somatic cell count in milk.

#### 2.4.2. Bacteriological Analysis

Identification of mastitis pathogens was performed for descriptive purposes only, in order to characterize IMIs present before dry-off and after calving, without using bacteriological results in the classification algorithm for dry-off therapy.

Milk samples were initially inoculated onto chromogenic, selective, and differential media for the isolation of major mastitis pathogens: ChromID^®^ CPS^®^ Elite agar (bioMérieux, Marcy-l’Étoile, France) for the detection and differentiation of coliforms and enterococci, and Baird-Parker agar (bioMérieux, Marcy-l’Étoile, France) for the selective isolation of *Staphylococcus aureus*. Colonies showing limited growth or small size on CPS agar were subsequently subcultured onto Columbia Blood Agar (bioMérieux, Marcy-l’Étoile, France), a nutrient-rich medium, to allow full bacterial development and evaluation of colony morphology and hemolysis patterns. Plates were incubated aerobically at 37 °C for 18–24 h to obtain isolated colonies.

Culture purity was assessed by morphological examination and Gram staining, and representative colonies were selected for identification. A sample was considered positive for IMI when ≥100 colony-forming units (CFU)/mL of any relevant pathogen were isolated, except for non-aureus staphylococci (NAS), for which a threshold of ≥200 CFU/mL was applied, according to the criteria described by Dohoo et al. [51] for the interpretation of single milk samples. Samples yielding three or more bacterial types were classified as contaminated, and the isolation of *Bacillus* spp. was considered non-significant. However, when *Staphylococcus aureus* was detected in a contaminated sample, the result was considered relevant and the sample was classified as positive, in accordance with the recommendations of Reyher et al. [52]. From pure cultures, bacterial suspensions were prepared in sterile saline solution (0.45–0.50% NaCl) and adjusted to a 0.5 McFarland standard (~1.5 × 10^8^ CFU/mL) using the DensiCHEK system (bioMérieux, Marcy-l’Étoile, France). Standardized suspensions were inoculated into appropriate VITEK^®^ 2 cards for Gram-positive or Gram-negative microorganisms and processed automatically using the VITEK^®^ 2 Compact system (bioMérieux, Marcy-l’Étoile, France), according to the manufacturer’s instructions and IDF standards for bacterial identification.

### 2.5. Dry-Off Treatment Protocol

Following classification of cows into risk categories based on SCC and DSCC values, the corresponding treatment protocol was applied. In the prospective study, 25 cows were classified as high-risk and 62 cows as low-risk. High-risk cows from the prospective cohort (*n* = 25), as well as low-risk cows from the historical cohort (*n* = 37), were treated at dry-off with intramammary antibiotics using Ubrostar^®^ (Hipra, Girona, Spain), a broad-spectrum combination containing penethamate hydriodide (100 mg), benethamine penicillin (280 mg), and framycetin sulfate (100 mg), administered in each quarter. ITS were not combined with intramammary antibiotics in high-risk cows. This design was selected because the primary objective of the study was not to evaluate the additive effect of teat sealants on antimicrobial dry cow therapy, but rather to assess whether low-risk cows could be managed safely without antibiotic treatment at dry-off. Therefore, the study compared an ITS-only strategy in low-risk cows with the conventional antimicrobial dry-off approach applied under local field conditions. In addition, the protocol reflected routine management practices in the participating farms, where internal teat sealants are not routinely combined with antimicrobial dry cow therapy.

All treatments were administered under strict aseptic conditions, given the recognized importance of application hygiene during intramammary dry cow treatment and teat sealant administration. The teat end of each quarter was disinfected using sterile gauze soaked in 70% ethanol and allowed to dry, after which the antibiotic or teat sealant was infused into the teat cistern. In the case of antibiotic administration, the product was gently massaged to facilitate its distribution within the mammary quarter. Following treatment, all teats were dipped in a post-milking disinfectant solution based on lactic acid and chlorhexidine (Kerba Dip Lacto^®^, Kerbl, Buchbach, Germany). Milk sampling and treatment administration at dry-off were performed by the study personnel. All procedures were carried out by the principal investigator to ensure strict adherence to the experimental protocol, aseptic conditions, and uniformity of treatment application. Formal blinding of treatment administration was not feasible under field conditions because the dry-off protocols differed between groups and were applied by study personnel. However, laboratory personnel performing milk analyses and farm staff recording clinical mastitis cases were not informed about treatment allocation, thereby reducing the risk of observer bias in outcome assessment.

### 2.6. Outcomes and Variables

To evaluate the impact of the dry-off treatment strategy, two groups of low-risk cows were compared: cows treated with intramammary antibiotics at dry-off (LR-AB) and cows treated exclusively with an ITS (LR-ITS). Analyses focused on udder health parameters, postpartum milk production, and factors potentially associated with these outcomes.

Farm was included as a categorical variable with two levels (F1 and F2). Age was expressed in months (Age_mo) and categorized into three groups (≤48, 49–72, and ≥73 months). Parity was recorded as the number of calvings and grouped into three categories (parity 1, parity 2–3, and parity ≥ 4). Dry period length (DPL) was expressed in days and categorized into two groups (45–65 days and >65 days).

#### 2.6.1. Udder Health

SCC was log-transformed into somatic cell score (SCS) using the standard formula SCS = log_2_(SCC/100,000) + 3 [53] to improve normality and allow the use of parametric statistical methods. SCS was calculated as the mean value over the last three months prior to dry-off (SCS_preDO), at dry-off (SCS_DO), and at the first postpartum test (SCS_PP). Postpartum SCS was analyzed both as a continuous variable and as a categorical variable using the conventional threshold of 200,000 cells/mL (SCS = 4). Differential somatic cell count (DSCC) was evaluated at dry-off (DSCC_DO) and at the first postpartum test (DSCC_PP). Postpartum DSCC was analyzed both as a continuous variable and as a categorical variable using a threshold of 65% (≤65% and >65%).

Intramammary infection at dry-off (IMI_DO) and postpartum (IMI_PP) was classified into three categories: absent, major pathogen, and minor pathogen. When both major and minor pathogens were isolated, the infection was classified as major. For the purposes of this study, postpartum IMIs referred to intramammary infections detected at the first postpartum sampling. In low-risk cows considered uninfected at dry-off, these infections were interpreted as likely acquired during the dry period or shortly after calving, although persistent subclinical infections cannot be entirely excluded. CM was defined as the presence of visible milk abnormalities (flakes, clots, or watery secretion) and/or local signs of mammary gland inflammation, as observed and reported by farm personnel or study personnel when present. CM occurring within the first 100 days in milk (DIM) was included as a binary outcome variable. In addition, time to first CM event (CM_DIM) was recorded and used for time-to-event analysis. The main comparative analyses included only low-risk cows.

#### 2.6.2. Milk Yield

Milk production prior to dry-off was assessed using two variables: the average milk yield during the last three months before dry-off (MY_preDO, L/day), categorized based on the median value (22.63 L/day), and milk yield at the time of dry-off (MY_DO, L/day), defined as milk production recorded at the last milking before dry-off and categorized using a median cutoff of 5 L/day.

Test-day records within the first 100 days in milk (DIM) were used to evaluate performance. Each record included DIM, milk yield (MY, L/day), and SCS. Mean milk yield (MY_100DIM_mean) and mean SCS (SCS_100DIM_mean) over the first 100 DIM were calculated as the arithmetic mean of all available test-day records.

#### 2.6.3. Antimicrobial Use

AMU was evaluated in low-risk cows by comparing the LR-AB and LR-ITS groups. Total antimicrobial consumption was calculated as the sum of antimicrobials administered at dry-off and those used for treatment of CM during the first 100 DIM. At dry-off, antimicrobial use was calculated based on the number of syringes administered per quarter and the active substance content of Ubrostar^®^ (319.8 mg per syringe), resulting in a total of 1279.2 mg per cow. CM was treated using Ubrolexin^®^ (Boehringer Ingelheim, Ingelheim am Rhein, Germany), containing cefalexin (200 mg) and kanamycin (100 mg equivalent), corresponding to 300 mg of active substance per syringe. The treatment protocol consisted of one syringe per day for three consecutive days, resulting in a total AMU of 900 mg per mastitis case. No systemic antimicrobial treatments were administered either at dry-off or for mastitis cases during the study period. Therefore, antimicrobial use calculations included all antimicrobials administered to enrolled cows. Total AMU was expressed both at group level (mg/group) and standardized per cow (mg/cow). Additionally, the potential AMU under a BDCT scenario was estimated by multiplying the number of low-risk cows by the antimicrobial amount administered per cow at dry-off.

#### 2.6.4. Economic Analysis

An economic analysis was performed to evaluate the financial impact of using internal teat sealants at dry-off compared with intramammary antibiotic treatment in low-risk cows. Costs were calculated from the farmer’s perspective and included dry-off treatment costs, costs associated with clinical mastitis cases occurring within the first 100 DIM, and economic losses due to discarded milk during the antimicrobial withdrawal period. Costs associated with mastitis-related culling were not included, as no such cases occurred during the study period. Potential reductions in milk yield attributable to clinical mastitis were also not incorporated into the analysis, because the available data did not allow reliable estimation of production losses specifically caused by mastitis. Therefore, the economic model focused on directly documented costs.

All costs were initially calculated in Romanian lei (RON) and subsequently converted to euros (€) using an exchange rate of 1 € = 5.09 RON, corresponding to the average exchange rate during the study period. Dry-off treatment costs were based on farm-level purchase prices. For the antibiotic-based strategy, the cost of intramammary therapy was 14.80 €/cow (four syringes per cow), whereas the cost for the internal teat sealant-only strategy was 10.92 €/cow. Costs associated with CM included both treatment expenses and economic losses due to discarded milk. In all recorded CM cases, antimicrobial treatment was administered. Therefore, discarded milk losses were calculated according to the antimicrobial withdrawal period, which encompassed the period during which milk was withheld because of both antimicrobial residues and clinical abnormalities. Milk discarded solely due to clinical signs in the absence of antimicrobial treatment was not calculated separately, as no untreated CM cases occurred during the study. Mastitis treatment consisted of intramammary administration of Ubrolexin^®^ for three consecutive days, combined with systemic anti-inflammatory treatment using ketoprofen. The total treatment cost was estimated at 23.27 €/case. The quantity of discarded milk was calculated based on the daily milk yield recorded at the closest test-day to the occurrence of mastitis. Daily MY was multiplied by the withdrawal period of the antimicrobial used (5 days; 10 milkings), resulting in the total volume of discarded milk. Economic losses were calculated using the average milk price paid by the cooperative (0.47 €/L).

The total economic cost per cow was calculated using the following equation:

(1) $$\;\;C_{total/cow}=\;C_{dry\text{-}off/cow}+\;\frac{C_{mastitis}+\sum (MYᵢ\times WP\times P_{milk})}{N}$$

where

$C_{total/cow}$ = total economic cost per cow (€).

$C_{dry\text{-}off/cow}$ = dry-off treatment cost per cow (€).

$C_{mastitis}$ = total cost of mastitis treatments at group level for clinically affected cows (€).

MYᵢ = estimated daily milk yield of mastitic cow *i* (L/day).

WP = antimicrobial withdrawal period (days).

$P_{milk}$ = milk price (€/L).

N = total number of cows in the group.

The term $MYᵢ\times WP\times P_{milk}$ represents the economic loss associated with discarded milk during the antimicrobial withdrawal period.

Costs associated with the classification of cows were calculated separately. Classification was based on the determination of SCC and DSCC values, with the total cost of laboratory analyses estimated at 1 € per cow. As these costs were incurred at the level of the entire prospective cohort and could not be attributed exclusively to low-risk cows, they were not included in the main economic comparison between the LR-AB and LR-ITS groups, but were reported separately as implementation costs of the SDCT strategy.

The economic input parameters used in the analysis are summarized in Table 1.

An additional herd-level stratified economic analysis was performed to explore the influence of farm management conditions and herd infection status on the economic performance of the SDCT strategy.

### 2.7. Statistical Analysis

Statistical analyses were performed using R software version 4.5.3 (R Foundation for Statistical Computing, Vienna, Austria). The primary analysis focused on cows classified as low-risk at dry-off, treated either with intramammary antibiotics or ITS. High-risk cows were excluded from comparative analyses. Continuous variables were assessed for distribution and are presented as median and interquartile range (IQR), while categorical variables are reported as absolute frequencies and percentages. Group comparisons (LR-ITS vs. LR-AB) were performed using the Wilcoxon rank-sum test for continuous variables and Fisher’s exact test for categorical variables. Multivariable regression models were used to evaluate the effect of treatment on postpartum outcomes. Linear regression models were applied for continuous outcomes, including SCS_PP, DSCC_PP, MY_100DIM_mean, and SCS_100DIM_mean. Binary outcomes, including elevated postpartum SCS (SCS_PP ≥ 4), elevated DSCC (>65%), presence of IMI_PP, and occurrence of CM within the first 100 DIM, were analyzed using logistic regression models. Results are reported as odds ratios (OR) with 95% confidence intervals (95% CI).

Multivariable models were adjusted for potential confounders selected a priori based on biological relevance, including farm, age group, parity group, MY_preDO, MY_DO, SCS_DO, DSCC_DO, and DPL_G. IMI_DO was not included due to lack of variability within the low-risk population.

To assess whether treatment effects differed between farms, an interaction term between treatment and farm was included. Additionally, descriptive analyses stratified by farm were performed.

Time-to-event analysis for CM was conducted using Cox proportional hazards models, with results presented as hazard ratios (HR) and 95% confidence intervals. Kaplan–Meier curves were used to illustrate mastitis-free survival, and group comparisons were performed using the log-rank test. Given the limited number of events for some outcomes, parsimonious models were used to avoid overfitting. In cases of quasi-complete or complete separation, analyses were conducted descriptively and using Fisher’s exact test.

Statistical significance was set at *p* < 0.05.

Antimicrobial use and economic outcomes were calculated using a deterministic, descriptive approach based on predefined unit costs and recorded treatment data. No inferential statistical models were applied for these outcomes. Results are therefore presented descriptively as total values and averages per cow.

### 2.8. Ethical Approval

The study protocol was approved by the Bioethics Committee of the University of Agricultural Sciences and Veterinary Medicine of Cluj-Napoca (approval no. 514/10.04.2025), in accordance with applicable national and European legislation on animal research ethics.

## 3. Results

A total of 99 low-risk cows were included in the study, of which 37 received intramammary antibiotics (LR-AB) and 62 received an internal teat sealant (LR-ITS). Baseline characteristics were generally comparable between the two groups (Table 2). However, cows in the LR-ITS group were older (median: 56 vs. 45 months; *p* = 0.005) and had a higher proportion of animals in the ≥73 months category (24% vs. 11%).

Parity distribution also differed between groups (*p* = 0.027), with a greater proportion of cows with parity ≥ 4 in the LR-ITS group. No significant differences were observed between groups in milk yield prior to dry-off, somatic cell score, or differential somatic cell count at dry-off.

### 3.1. Udder Health

#### 3.1.1. Postpartum IMIs

The proportion of cows without postpartum IMIs was comparable between treatment groups, being 78.4% in the LR-AB group and 75.8% in the LR-ITS group.

The prevalence of major pathogens was low in both groups (5.4% in the antibiotic group vs. 6.5% in the ITS group). Similarly, the proportion of minor pathogen infections was comparable between groups (16.2% vs. 17.7%).

Major pathogens identified postpartum included *Staphylococcus aureus*, *Streptococcus uberis*, *Streptococcus dysgalactiae*, and *Escherichia coli*, although their overall prevalence remained low in both treatment groups. Minor pathogens consisted predominantly of NAS, including *Staphylococcus chromogenes*, *S. xylosus*, *S. simulans*, *S. epidermidis*, and *S. haemolyticus*, together with *Corynebacterium bovis*. No fungal, yeast, or algal isolates were detected.

**Figure 2:**
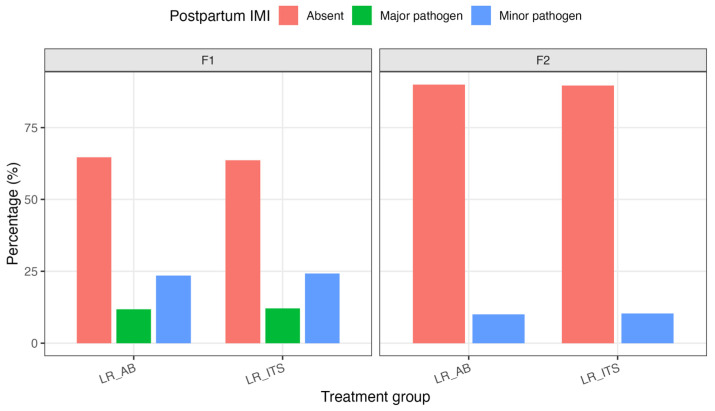
Distribution of postpartum IMI status by treatment group and farm. The figure illustrates the distribution (%) of cows classified as uninfected (Absent), infected with major pathogens, or infected with minor pathogens at the first postpartum milk recording. Low-risk cows were treated at drying-off either with intramammary antibiotics (LR_AB) or with an ITS (LR_ITS). Results are presented separately for each farm (F1 and F2). In both farms, the distribution of IMI categories was similar between treatment groups, with no evident differences in the proportion of infected cows. Farm-related differences are visible, with a lower overall prevalence of infection and absence of major pathogens in F2 compared to F1.

Multivariable analysis did not reveal a significant effect of treatment on the risk of postpartum IMI (OR = 0.94; 95% CI: 0.31–2.99; *p* = 0.92). In contrast, farm had a significant effect on infection risk, with cows in farm F2 showing a lower risk compared to F1 (OR = 0.20; 95% CI: 0.05–0.65; *p* = 0.01). Additionally, a DPL greater than 65 days was associated with an increased risk of postpartum IMI (OR = 3.53; 95% CI: 1.21–10.87; *p* = 0.02). Farm-stratified analysis confirmed these findings, with no evident differences between treatment groups. In F1, the proportion of uninfected cows was similar between groups (64.7% vs. 63.6%), and the distribution of major and minor pathogens was comparable. In F2, the overall infection prevalence was lower, with no cases of major pathogens and similar proportions of minor infections between treatments (10.0% vs. 10.3%). The distribution of postpartum IMI categories by treatment group and farm is illustrated in Figure 2.

#### 3.1.2. Somatic Cell Indicators (SCS and DSCC)

SCS and DSCC were evaluated as indicators of udder health in low-risk cows, both at the first postpartum milk recording and SCS during the first 100 DIM.

**Figure 3:**
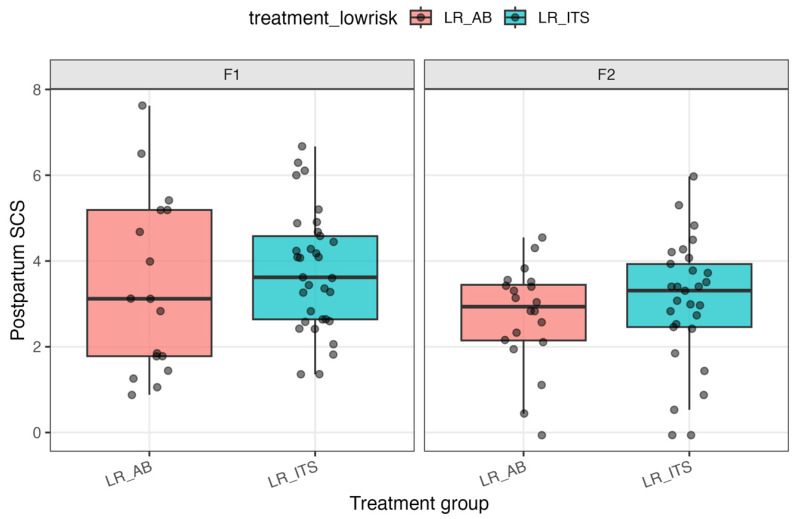
SCS at the first postpartum milk recording in low-risk cows by treatment group and farm. Boxplots illustrate the distribution of SCS at the first postpartum milk recording in low-risk cows treated at drying-off with either intramammary antibiotics (LR_AB) or ITS (LR_ITS). Data are presented separately for each farm (F1 and F2). The central line represents the median, boxes indicate the interquartile range (IQR), whiskers represent the range, and points correspond to individual observations. No evident differences in SCS distribution between treatment groups are observed in either farm.

At the first postpartum test, SCS values showed a similar distribution between treatment groups (LR-AB vs. LR-ITS) within both farms (Figure 3). Median SCS values were slightly higher in the LR-ITS group compared with LR-AB in both F1 and F2; however, substantial overlap in interquartile ranges indicated no clear separation between treatments.

In the multivariable linear model restricted to low-risk cows, treatment at dry-off was not significantly associated with postpartum SCS (β = 0.25, *p* = 0.37). In contrast, higher SCS at dry-off was strongly associated with increased postpartum SCS (β = 0.60, *p* < 0.001). Additionally, cows aged 49–72 months had significantly lower SCS compared with younger cows (*p* = 0.005).

Logistic regression analysis of elevated postpartum SCS yielded similar results. Treatment with ITS (LR-ITS) was not associated with increased odds of high SCS (OR = 1.86, *p* = 0.26), whereas cows from farm F2 had significantly lower odds compared with F1 (OR = 0.15, *p* = 0.0015). Higher SCS at dry-off was strongly associated with increased odds of elevated postpartum SCS (OR = 3.44, *p* < 0.001).

**Figure 4:**
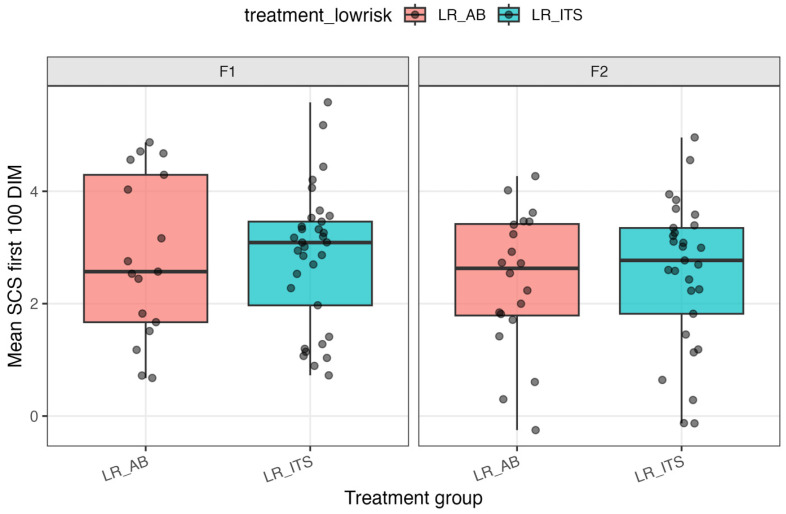
Mean somatic cell score during the first 100 days in milk in low-risk cows by treatment group and farm. Boxplots represent the distribution of mean SCS during the first 100 DIM in low-risk cows treated at drying-off with either intramammary antibiotics (LR_AB) or ITS (LR_ITS). Results are shown separately for each farm (F1 and F2). The central line represents the median, boxes indicate the interquartile range (IQR), whiskers represent the range, and points correspond to individual observations. Similar distributions of SCS are observed between treatment groups across both farms.

During the first 100 DIM, mean SCS did not differ significantly between treatment groups in either farm (Figure 4). The distribution of SCS values remained comparable between the LR-AB and LR-ITS groups.

In the adjusted linear model, treatment at dry-off was not associated with mean SCS during early lactation (β = 0.02, *p* = 0.94). As observed for SCS at the first postpartum test, SCS at dry-off remained a strong predictor of SCS during early lactation (β = 0.45, *p* < 0.001). Additionally, longer dry periods (>65 days) were associated with higher SCS values (*p* = 0.018).

**Figure 5:**
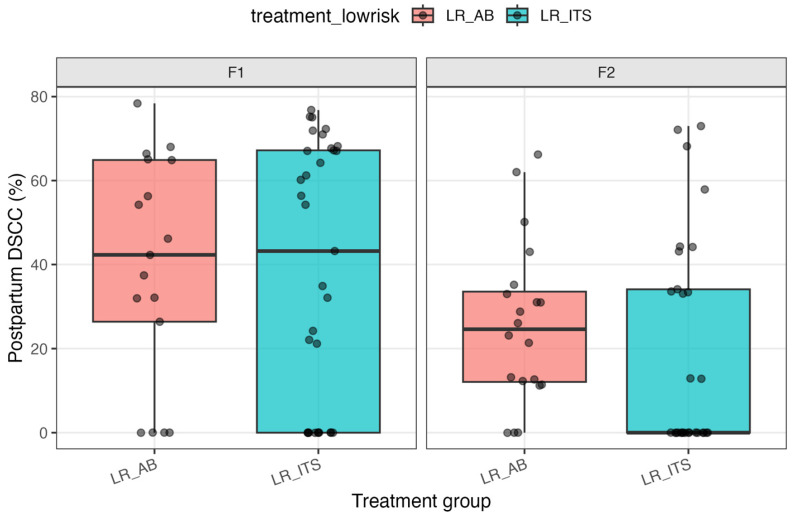
DSCC at the first postpartum milk recording in low-risk cows by treatment group and farm. Boxplots illustrate the distribution of DSCC (%) at the first postpartum milk recording in low-risk cows treated at drying-off with either intramammary antibiotics (LR_AB) or ITS (LR_ITS). Results are presented separately for each farm (F1 and F2). The central line represents the median, boxes indicate the interquartile range (IQR), whiskers represent the range, and points correspond to individual observations. High variability of DSCC values is observed within both treatment groups, particularly in the LR_ITS group, where a higher proportion of zero values is evident. Overall, no clear differences between treatment groups are observed within farms.

DSCC values at the first postpartum test showed high variability within both treatment groups, particularly in the LR-ITS group, where a notable proportion of cows had DSCC values equal to zero (Figure 5). Despite this variability, no consistent differences between treatment groups were observed within farms. Although some visual differences in DSCC distribution were apparent, particularly within the LR-ITS group, these patterns were not supported by statistical significance, likely reflecting substantial within-group variability and the relatively limited sample size.

In the linear model, treatment at dry-off was not significantly associated with DSCC values (*p* = 0.33). However, higher milk yield at dry-off (>5 L/day) was associated with increased DSCC postpartum (*p* = 0.007), and higher SCS at dry-off was also positively associated with DSCC (*p* = 0.045). Longer dry periods (>65 days) were strongly associated with higher DSCC values (*p* < 0.001).

Logistic regression analysis of elevated postpartum DSCC yielded similar results, with no significant effect of treatment (OR = 1.89, *p* = 0.33). A significant farm effect was observed, with cows in F2 having lower odds of elevated DSCC compared with those in F1 (OR = 0.24, *p* = 0.035). Additionally, longer dry periods (>65 days) were associated with increased odds of high DSCC (OR = 3.38, *p* = 0.042).

Overall, no significant differences were observed between treatment groups (LR_AB vs. LR_ITS) for SCS or DSCC, either at the first postpartum test or during early lactation. Instead, indicators of udder health were primarily influenced by cow-level factors, particularly SCS at dry-off and DPL, as well as by farm-specific effects.

#### 3.1.3. CM During the First 100 DIM

The occurrence of CM during the first 100 DIM was low, with a total of 17 events recorded out of 99 animals. In the multivariable logistic regression model, treatment at dry-off was not associated with CM occurrence. Cows treated with ITS (LR-ITS) had similar odds of developing CM compared with those treated with intramammary antibiotics (LR-AB) (OR = 1.05; 95% CI: 0.34–3.46; *p* = 0.94).

**Figure 6:**
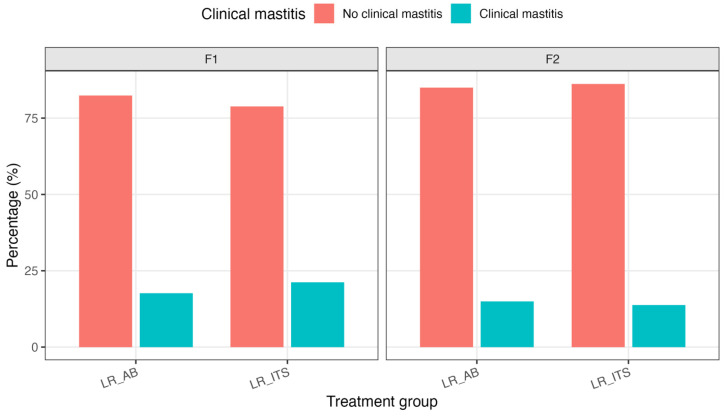
Clinical mastitis incidence during the first 100 DIM, stratified by treatment group (intramammary antibiotics, LR_AB; ITS, LR_ITS) and farm (F1 and F2). Bars represent the percentage of cows with and without CM in each group. Differences between groups were small and consistent with the results of multivariable analyses showing no significant effect of treatment on CM occurrence.

No significant effect of farm was observed (OR for F2 vs. F1 = 0.95; 95% CI: 0.29–3.10; *p* = 0.93). Similarly, prepartum udder health indicators, SCS and DSCC at dry-off, were not associated with CM occurrence. A longer dry period (>65 days) showed a tendency toward increased odds of CM (OR = 2.48; 95% CI: 0.79–8.06), although this association was not statistically significant (*p* = 0.12). Time-to-event analysis using a Cox proportional hazards model confirmed the absence of a treatment effect on CM risk. The hazard of developing CM did not differ between treatment groups (HR = 1.08; 95% CI: 0.40–2.92; *p* = 0.88). Farm was also not significantly associated with time to CM (HR for F2 vs. F1 = 0.66; 95% CI: 0.25–1.75; *p* = 0.41). Descriptive analysis showed similar proportions of CM between treatment groups in both farms, as illustrated in Figure 6.

### 3.2. Milk Yield

**Figure 7:**
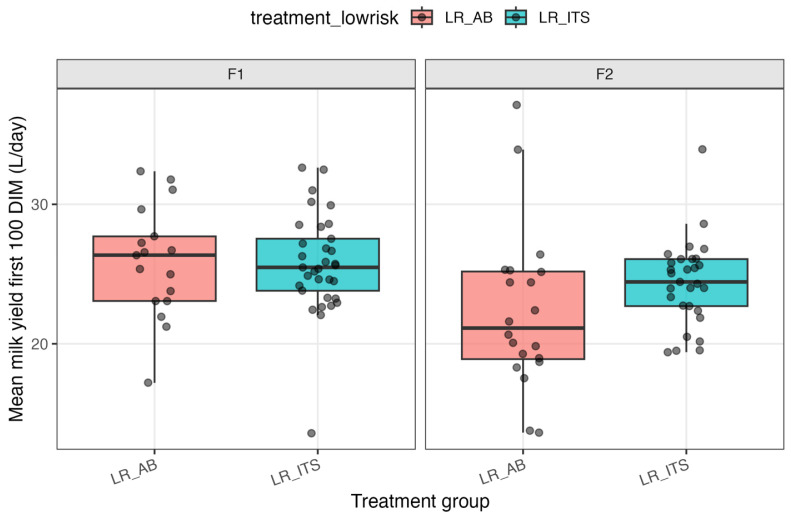
MY during the first 100 DIM. Distribution of mean milk yield during the first 100 DIM according to treatment group (intramammary antibiotics, LR_AB; ITS, LR_ITS) and farm (F1 and F2). Boxplots represent median, interquartile range, and individual observations. Although numerical differences were observed between farms, no statistically significant effect of treatment on MY was identified in the adjusted linear models.

Mean milk yield during the first 100 DIM was similar between treatment groups in both descriptive and adjusted analyses (Figure 7). In farm F1, median milk yield appeared slightly higher in cows treated with intramammary antibiotics (LR-AB) compared with those treated with internal teat sealant (LR-ITS), whereas in farm F2, cows in the LR-ITS group showed numerically higher MY. In the multivariable linear model, treatment with ITS (LR-ITS) was not significantly associated with MY compared with antibiotic treatment (β = −0.56 L/day; 95% CI: −1.94 to 0.82; *p* = 0.42). MY was significantly associated with age, with older cows producing more milk compared with younger animals (49–72 months: β = 3.65 L/day, *p* < 0.001; ≥73 months: β = 3.84 L/day, *p* = 0.001). Higher pre-dry-off milk yield (>22.63 L/day) was also positively associated with milk production in early lactation (β = 3.75 L/day; *p* < 0.001). No significant associations were observed for SCS_DO, DSCC_DO, or DPL.

### 3.3. Antimicrobial Use

In the LR-AB group (*n* = 37), AMU associated with dry-off treatment amounted to 47,330.4 mg per group, corresponding to 1279.2 mg per cow. During the first 100 DIM, the six CM cases required an additional 5400 mg of antimicrobials, resulting in a total AMU of 52,730.4 mg per group (1425.1 mg per cow). In the LR-ITS group (*n* = 62), no antimicrobials were administered at dry-off. The 11 CM cases recorded during the first 100 DIM resulted in a total AMU of 9900 mg (159.7 mg per cow).

Overall, the ITS-only strategy applied to low-risk cows was associated with an 88.8% reduction in antimicrobial use per cow compared with intramammary antibiotic treatment at dry-off.

In the prospective cohort, 62 out of 87 cows (71.3%) were classified as low-risk and therefore did not receive antimicrobials at dry-off. In the absence of SDCT, these animals would have been treated according to a BDCT approach, resulting in an estimated additional use of 79,310.4 mg of antimicrobial active substance at dry-off. Detailed AMU data are presented in Table 3.

### 3.4. Economic Outcomes

In the LR-AB group (*n* = 37), the cost of dry-off treatment was 14.81 €/cow. During the first 100 DIM, six cases of CM resulted in total economic losses of 444.8 € per group, including both treatment costs and losses associated with discarded milk. These losses corresponded to a mean mastitis-related cost of 12.02 €/cow and a total economic cost of 26.83 €/cow.

In the LR-ITS group (*n* = 62), the cost of dry-off treatment was 10.92 €/cow. The 11 CM cases recorded during the first 100 DIM resulted in total losses of 920.4 € per group, corresponding to a mastitis-related cost of 14.84 €/cow. The total economic cost in this group was 25.76 €/cow. Detailed economic outcomes are presented in Table 4.

Differences between farms were observed in the economic performance of the treatment strategies. In farm F1, the total cost per cow was 27.63 € for the LR-AB group and 28.62 € for the LR-ITS group, indicating a slightly higher cost of the sealant-based strategy under conditions of higher postpartum mastitis incidence. In farm F2, the total cost per cow was 25.32 € for the LR-AB group and 22.50 € for the LR-ITS group, suggesting a better economic performance of the ITS strategy in this herd. Herd-level economic outcomes are summarized in Table 5.

**Figure 8:**
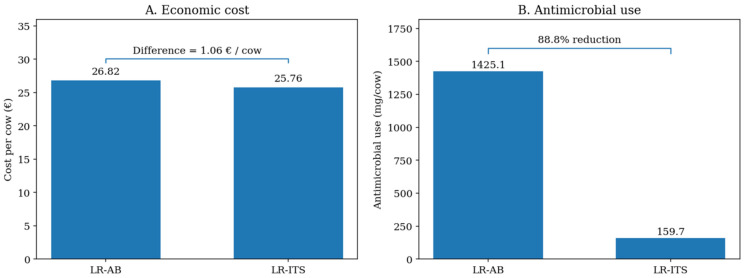
Comparison of economic costs and antimicrobial use between low-risk cows treated with intramammary antibiotics at dry-off (LR-AB) and low-risk cows treated exclusively with an internal teat sealant (LR-ITS). Panel (**A**) shows the total economic cost per cow, including dry-off treatment and mastitis-related costs during the first 100 DIM. Panel (**B**) shows the total AMU per cow (mg of active substance), including antimicrobials used at dry-off and for treatment of CM during the first 100 DIM. While economic costs were similar between strategies, the ITS-only approach was associated with a substantial reduction in AMU.

Overall, economic costs were similar between treatment strategies, as illustrated in Figure 8, despite the substantial reduction in antimicrobial use observed in the LR-ITS group.

## 4. Discussion

The results of the present study showed that, in low-risk cows, the use of ITS at dry-off was not associated with an increased risk of postpartum IMIs, elevated SCS or DSCC values, or CM, compared with intramammary antibiotic treatment. The absence of significant differences between the LR-ITS and LR-AB groups suggests that antibiotic administration in healthy cows at dry-off does not provide additional benefits in terms of udder health. These findings support the principles of SDCT, indicating that low-risk cows can be safely managed without antibiotics when appropriate selection criteria and ITS are used.

The evolution of research has shown that, although early studies favored BDCT [8,13,14,54,55,56,57], under current conditions characterized by improved selection criteria and the use of ITS, SDCT provides comparable outcomes in terms of udder health. For example, Cameron et al. [43] found no significant differences in new IMI incidence, while reporting a higher incidence of fungal infections in antibiotic-treated cows, highlighting potential unintended consequences of routine antimicrobial use. Similarly, several studies have shown that, when selection is properly applied, SDCT provides a level of protection comparable to BDCT, particularly in herds with low infection pressure [4,5,26,30,31,35,42], and in some cases may even be associated with lower SCC values [41]. In addition, large-scale implementation of SDCT in the Netherlands was not associated with significant changes in herd-level SCC or CM incidence [44]. The findings of the present study are consistent with this body of evidence and further support the safe omission of antibiotics in low-risk cows. However, as highlighted by our results, the effectiveness of this approach is strongly influenced by farm-specific conditions and management practices. Differences observed between farms, particularly in infection prevalence and economic performance, emphasize that the success of SDCT is not solely dependent on treatment choice, but also on the level of infection pressure, hygiene, and overall herd management. This also applies to ITS-based dry-off strategies, whose safety and effectiveness depend on proper application technique, as unhygienic administration may introduce pathogens into the teat canal and increase the risk of new IMIs. Therefore, the present findings should not be interpreted as supporting universal omission of antimicrobials or indiscriminate use of teat sealants, but rather as evidence generated under controlled application conditions and in herds with favorable udder health status and management practices. This observation is consistent with previous studies showing that SDCT outcomes may vary considerably between farms depending on factors such as baseline mastitis prevalence, udder health status, hygiene standards, and dry period management practices [19].

Although combining SCC and DSCC may improve sensitivity for detecting intramammary inflammation, this may occur at the expense of reduced specificity and potentially greater antimicrobial use, as previously reported in the literature [25,58]. In the present study, the SCC–DSCC combination was used as a conservative decision-support approach aimed at minimizing the risk of leaving potentially infected cows untreated at dry-off rather than as a formally validated diagnostic test. Whether DSCC provides substantial additional discriminatory value beyond SCC alone remains an area requiring further investigation.

Milk production during the first 100 DIM was not significantly affected by treatment, indicating that omitting antibiotics in low-risk cows does not compromise productive performance. This is in line with previous studies demonstrating that SDCT does not negatively affect MY when cows are appropriately selected [34,59], and that comparable production levels can be achieved between SDCT and BDCT when ITS are used [26,27,30,35]. Given that IMI present at calving can reduce MY by approximately 5% [60], maintaining udder health during the dry period remains critical. While antimicrobial dry cow therapy is primarily intended to eliminate existing infections acquired during lactation, our results suggest that, in appropriately selected low-risk cows, comparable postpartum udder health can be maintained without routine antibiotic administration at dry-off.

At the individual level, cow-related factors had a greater influence on udder health and production outcomes than treatment type. In particular, SCS at dry-off was a strong predictor of postpartum SCS, while MY and age influenced production during early lactation. These findings are consistent with the literature highlighting the importance of individual factors such as MY, parity, and age in determining IMI risk and udder health dynamics [14,23,34,61,62,63,64]. In addition, dry period length was associated with variations in inflammatory indicators, supporting previous reports linking extended dry periods with altered udder health outcomes [14,23,65]. This relationship is likely multifactorial and may involve not only dry period exposure but also metabolic and management-related factors, including changes in energy balance and transition cow physiology [66]. Together, these results reinforce the concept that SDCT outcomes are driven by a complex interaction between animal-level factors and management conditions.

A major advantage of the ITS-only strategy observed in this study was the substantial reduction in AMU. The omission of antibiotics in low-risk cows resulted in an 88.8% reduction in AMU per cow, a value at the upper range reported in the literature. Similar reductions have been documented in previous studies, ranging from approximately 20% to over 80% depending on selection criteria and herd characteristics [27,31,35,41,42,67]. The magnitude of reduction observed in this study reflects the high proportion of low-risk cows (71.3%) and suggests that, in herds with good mastitis control, a large proportion of animals can be managed without antibiotics. These findings also support concerns raised in the literature regarding the inefficient use of antimicrobials under BDCT, where a large proportion of treated quarters may not require antibiotic therapy [13,21]. Importantly, the variability in antimicrobial reduction reported across studies further highlights the influence of herd-level factors and management practices on SDCT outcomes.

From an economic perspective, the ITS-only strategy resulted in total costs comparable to antibiotic treatment. Although treatment costs at dry-off were lower in the LR-ITS group, differences in total economic outcomes were primarily driven by the incidence of CM and associated milk withdrawal losses. The herd-level analysis demonstrated variability in economic performance between the two enrolled farms. In one herd, the sealant-based strategy was associated with slightly higher total costs, whereas in the other herd it appeared economically more favorable. Although only two farms were included and infection pressure was not formally characterized, these findings suggest that farm-specific management conditions and epidemiological context may influence not only biological outcomes but also the economic performance of SDCT. Previous studies have reported variable economic outcomes of SDCT, largely depending on factors such as diagnostic accuracy, labor costs, antimicrobial prices, and infection prevalence [35]. Labor and time investment associated with SDCT implementation should also be considered. Although labor costs were not included in the present economic analysis, the process of identifying eligible cows based on SCC and DSCC may require additional time and data management compared with BDCT. However, under the evaluated protocol, the replacement of antimicrobial tubes with ITS in selected cows likely balanced much of the treatment-related workload. Some studies have shown modest economic benefits associated with SDCT [68], while others have highlighted its cost-effectiveness when selection algorithms are used [40] or when ITS are incorporated into the protocol [45]. It should also be considered that, in many contemporary dry cow therapy protocols, ITS are administered to all cows at dry-off, including those receiving antimicrobial treatment. In the present study, teat sealants were used exclusively in low-risk cows and were not combined with antimicrobials in high-risk animals, reflecting both local management practices and the objectives of the study design. Therefore, the economic comparison presented here reflects the specific treatment protocols evaluated and should not be interpreted as a direct comparison between ITS-only and antimicrobial-plus-ITS strategies. Under protocols where teat sealants are routinely combined with antimicrobial dry cow therapy, the relative economic advantage of the ITS-only strategy in low-risk cows could potentially be greater. Market conditions may also influence the optimal strategy, as higher milk prices may justify increased antibiotic use to prevent production losses, whereas lower prices favor antimicrobial reduction strategies [69]. Overall, both the present study and the available literature suggest that SDCT does not negatively impact farm profitability while contributing substantially to antimicrobial stewardship. Finally, it should be noted that the economic estimates presented here are specific to the conditions of the studied farms, including local prices for veterinary products and milk. As these parameters may vary considerably across regions and production systems, the direct extrapolation of these results should be made with caution.

The present study was designed as a pilot field study aimed at evaluating the feasibility and impact of implementing SDCT in dairy farms in Romania, where this approach is still rarely applied. In addition, the analysis focused exclusively on low-risk cows and did not include a direct comparison between SDCT and BDCT, which limits the extrapolation of the results at the herd level. The relatively small number of herds included limits the generalizability of the findings. Nevertheless, the present findings may be particularly relevant for Eastern European dairy systems, where selective dry cow therapy remains relatively underutilized and region-specific data are still limited. Differences in herd structure, breed composition, management practices, and adoption of milk recording systems may influence both the implementation and performance of SDCT, highlighting the need for additional studies under local production conditions. However, both farms were managed under comparable conditions and belonged to the same cooperative system, allowing the evaluation of SDCT implementation under relatively controlled field conditions. The use of a historical cohort enabled comparison with cows previously managed under conventional dry cow therapy within the same herds, thereby limiting some sources of confounding related to herd management, pathogen ecology, and veterinary practices. However, comparisons between different time periods may still be influenced by environmental and seasonal factors, including weather conditions and feed quality.

Therefore, further studies conducted on larger populations and under a wider range of management conditions, including direct comparisons between SDCT and BDCT, are needed to validate and extend these findings.

## 5. Conclusions

The present study showed that, in low-risk cows, the use of ITS alone at dry-off provided postpartum udder health and milk production outcomes comparable to intramammary antibiotic treatment. No significant differences were observed regarding postpartum IMIs, SCS, DSCC, CM, or MY during the first 100 DIM. At the same time, the ITS-only strategy resulted in a substantial reduction in AMU without a significant negative economic impact. These findings support the use of ITS as an effective preventive approach within SDCT protocols for appropriately selected low-risk cows and under suitable herd management conditions, highlighting its potential to reduce antimicrobial use while maintaining udder health, productive performance, and economic sustainability.

## Figures and Tables

**Table 1:** Economic input parameters used in the economic analysis.

Parameter	Description	Value (€)	Unit	Source
Internal teat sealant	Cost per syringe (Ubroseal Blue Dry Cow^®^)	2.73	€/syringe	Farm purchase price
Internal teat sealant treatment	Cost per cow (4 quarters)	10.92	€/cow	Calculated
Intramammary antibiotic (dry-off)	Cost per syringe (Ubrostar^®^)	3.70	€/syringe	Farm purchase price
Intramammary antibiotic treatment	Cost per cow (4 quarters)	14.81	€/cow	Calculated
Mastitis treatment (intramammary)	Ubrolexin^®^ cost per treatment course	11.19	€/case	Farm purchase price
Anti-inflammatory treatment	Ketoprofen treatment	12.07	€/case	Farm purchase price
Total mastitis treatment cost	Antibiotic + anti- inflammatory	23.26	€/case	Calculated
Milk price	Average milk price paid by cooperative	0.47	€/L	Cooperative records
Withdrawal period	Milk withdrawal period after treatment	5	days	Product label
SCC + DSCC analysis	Laboratory analysis cost per cow	1	€/sample	Laboratory fee
Currency conversion	Exchange rate used for conversion	1 € = 5.09 RON	–	Average exchange rate during the study period

This table presents the unit prices and economic parameters used for calculating the costs associated with dry-off treatment, mastitis treatment, discarded milk, and laboratory testing. All costs were initially recorded in Romanian lei (RON) and subsequently converted to euros (€) using an exchange rate of 1 € = 5.09 RON, corresponding to the average exchange rate during the study period.

**Table 2:** Baseline characteristics of low-risk cows according to treatment group, with between-group comparisons.

Characteristic	Overall *N* = 99 ^1^	LR-AB *N* = 37 ^1^	LR-ITS *N* = 62 ^1^	*p*-Value ^2^
F				0.5
F1	50 (51%)	17 (46%)	33 (53%)	
F2	49 (49%)	20 (54%)	29 (47%)	
Age_mo	50 (43, 68)	45 (38, 62)	56 (47, 72)	0.005
AG				0.044
≤48 months	41 (41%)	21 (57%)	20 (32%)	
49–72 months	39 (39%)	12 (32%)	27 (44%)	
≥73 months	19 (19%)	4 (11%)	15 (24%)	
parity				0.2
1	19 (19%)	12 (32%)	7 (11%)	
2	38 (38%)	14 (38%)	24 (39%)	
3	18 (18%)	5 (14%)	13 (21%)	
4	13 (13%)	4 (11%)	9 (15%)	
5	9 (9.1%)	2 (5.4%)	7 (11%)	
6	2 (2.0%)	0 (0%)	2 (3.2%)	
PG				0.027
Parity 1	19 (19%)	12 (32%)	7 (11%)	
Parity 2–3	56 (57%)	19 (51%)	37 (60%)	
Parity ≥ 4	24 (24%)	6 (16%)	18 (29%)	
MY_preDO	22.7 (20.4, 25.3)	21.6 (18.2, 28.9)	23.1 (21.5, 25.1)	0.2
MY_preDO_cat				0.6
≤22.63 L/day	58 (59%)	23 (62%)	35 (56%)	
>22.63 L/day	41 (41%)	14 (38%)	27 (44%)	
MY_DO	4.90 (3.90, 6.80)	4.90 (3.70, 6.50)	4.85 (4.10, 6.90)	0.2
MY_DO_cat				>0.9
≤5 L/day	62 (63%)	23 (62%)	39 (63%)	
>5 L/day	37 (37%)	14 (38%)	23 (37%)	
SCS_preDO	2.41 (1.67, 2.85)	2.37 (1.67, 2.74)	2.45 (1.67, 2.92)	0.6
SCS_DO	2.96 (1.91, 3.78)	2.83 (1.91, 3.77)	3.15 (1.97, 3.78)	0.6
DSCC_DO	12 (0, 43)	22 (0, 43)	0 (0, 42)	0.5
IMI_DO				
Absent	99 (100%)	37 (100%)	62 (100%)	
DPL	60.0 (55.0, 67.0)	56.0 (53.0, 66.0)	61.0 (56.0, 67.0)	0.083
DPL_G				0.3
45–65 days	63 (64%)	26 (70%)	37 (60%)	
>65 days	36 (36%)	11 (30%)	25 (40%)	
SCS_PP	3.31 (2.42, 4.24)	3.04 (1.85, 3.83)	3.40 (2.58, 4.27)	0.13
SCS_PP_G				0.11
SCS < 4	68 (69%)	29 (78%)	39 (63%)	
SCS ≥ 4	31 (31%)	8 (22%)	23 (37%)	
DSCC_PP	31 (0, 58)	31 (12, 50)	23 (0, 61)	0.6
DSCC_PP_G				0.3
DSCC ≤ 65%	80 (81%)	32 (86%)	48 (77%)	
DSCC > 65%	19 (19%)	5 (14%)	14 (23%)	
IMI_PP				>0.9
Absent	76 (77%)	29 (78%)	47 (76%)	
Major pathogen	6 (6.1%)	2 (5.4%)	4 (6.5%)	
Minor pathogen	17 (17%)	6 (16%)	11 (18%)	
CM				0.8
No clinical mastitis	82 (83%)	31 (84%)	51 (82%)	
Clinical mastitis	17 (17%)	6 (16%)	11 (18%)	
CM_DIM	100 (100, 100)	100 (100, 100)	100 (100, 100)	0.8
MY_100DIM_mean	25.0 (22.4, 26.7)	24.4 (20.1, 26.6)	25.2 (23.2, 26.7)	0.14
SCS_100DIM_mean	2.85 (1.71, 3.46)	2.57 (1.71, 3.47)	3.00 (1.82, 3.39)	0.7

^1^ n (%); Median (Q1, Q3). ^2^ Pearson’s Chi-squared test; Wilcoxon rank sum test; Fisher’s exact test; NA. Baseline characteristics of low-risk cows stratified by treatment group (intramammary antibiotics, LR_AB; internal teat sealant, LR_ITS). Continuous variables are presented as median (Q1, Q3), and categorical variables as number (percentage). *p*-values represent comparisons between treatment groups and were calculated using the Wilcoxon rank-sum test for continuous variables and Chi-squared or Fisher’s exact test for categorical variables, as appropriate. Only variables measured prior to treatment were included. Abbreviations: F = farm; Age_mo = age at dry-off (months); AG = age group at dry-off; parity = lactation number; PG = parity group; MY_preDO = milk yield prior to dry-off (L/day); MY_preDO_cat = pre–dry-off milk yield category; MY_DO = milk yield at dry-off (L/day); MY_DO_cat = milk yield at dry-off category; SCS_preDO = somatic cell score prior to dry-off; SCS_DO = somatic cell score at the last test prior to dry-off; DSCC_DO = differential somatic cell count (%) at the last test prior to dry-off; IMI_DO = intramammary infection status prior to dry-off; DPL = dry period length (days); DPL_G = dry period length group; SCS_PP = postpartum somatic cell score (5–7 DIM); SCS_PP_G = postpartum somatic cell score group; DSCC_PP = postpartum differential somatic cell count (%) (5–7 DIM); DSCC_PP_G = postpartum differential somatic cell count group; IMI_PP = postpartum intramammary infection status; CM = clinical mastitis during the first 100 DIM; CM_DIM = days in milk at clinical mastitis occurrence; MY_100DIM_mean = mean milk yield during the first 100 DIM (L/day); SCS_100DIM_mean = mean somatic cell score during the first 100 DIM.

**Table 3:** AMU associated with dry-off treatment and CM therapy during the first 100 DIM in low-risk cows (LR-AB, LR-ITS).

Group	*n*	AMU at Dry-Off (mg/group)	Postpartum AMU (mg/group)	Total AMU (mg/group)	Total AMU (mg/cow)
LR-AB	37	47,330.4	5400.0	52,730.4	1425.1
LR-ITS	62	0	9900.0	9900.0	159.7

AMU is presented as total antimicrobial amount per group (mg/group) and standardized per cow (mg/cow). Postpartum AMU represents antimicrobials administered for treatment of clinical mastitis cases within the first 100 DIM. Abbreviations: AMU—antimicrobial use; LR-AB—low-risk cows treated with intramammary antibiotics at dry-off; LR-ITS—low-risk cows treated with internal teat sealant only; DIM—days in milk.

**Table 4:** Economic outcomes in low-risk cows (LR-AB, LR-ITS).

Variable	LR-AB (*n* = 37)	LR-ITS (*n* = 62)
Dry-off treatment cost (€/cow)	14.81	10.92
Mastitis treatment cost (€/cow)	3.76	4.55
Milk withdrawal losses (€/cow)	8.26	10.29
Total mastitis-related cost (€/cow)	12.02	14.84
Total economic cost (€/cow)	26.83	25.76

Economic costs per cow were calculated as the sum of dry-off treatment costs and mastitis-related costs (mastitis treatment and discarded milk during the antimicrobial withdrawal period). Recorded CM incidence during the first 100 DIM was 16.2% (6/37) in LR-AB cows and 17.7% (11/62) in LR-ITS cows.

**Table 5:** Herd-level economic outcomes in low-risk cows (LR-ITS, LR-AB).

Herd	Group	Dry-Off Treatment Cost (€/cow)	Mastitis-Related Cost (€/cow)	Total Economic Cost (€/cow)
Herd 1	LR-AB (*n* = 17)	14.81	13.78	27.63
Herd 1	LR-ITS (*n* = 33)	10.92	17.70	28.62
Herd 2	LR-AB (*n* = 20)	14.81	10.51	25.32
Herd 2	LR-ITS (*n* = 29)	10.92	11.59	22.50

Herd-level economic outcomes in low-risk cows treated with intramammary antibiotics at dry-off (LR-AB) or ITS only (LR-ITS). Costs are expressed per cow (€) and include dry-off treatment costs and mastitis-related costs (treatment and discarded milk during the antimicrobial withdrawal period) during the first 100 DIM. Results are presented separately for each farm (F1 and F2).

## Data Availability

Data are available upon reasonable request.
